# Questions and controversies surrounding the perception and neural coding of pitch

**DOI:** 10.3389/fnins.2022.1074752

**Published:** 2023-01-09

**Authors:** Andrew J. Oxenham

**Affiliations:** ^1^Center for Applied and Translational Sensory Science, University of Minnesota Twin Cities, Minneapolis, MN, United States; ^2^Department of Psychology, University of Minnesota Twin Cities, Minneapolis, MN, United States

**Keywords:** pitch, auditory perception, auditory neuroscience, computational models, cochlear filtering, phase locking

## Abstract

Pitch is a fundamental aspect of auditory perception that plays an important role in our ability to understand speech, appreciate music, and attend to one sound while ignoring others. The questions surrounding how pitch is represented in the auditory system, and how our percept relates to the underlying acoustic waveform, have been a topic of inquiry and debate for well over a century. New findings and technological innovations have led to challenges of some long-standing assumptions and have raised new questions. This article reviews some recent developments in the study of pitch coding and perception and focuses on the topic of how pitch information is extracted from peripheral representations based on frequency-to-place mapping (tonotopy), stimulus-driven auditory-nerve spike timing (phase locking), or a combination of both. Although a definitive resolution has proved elusive, the answers to these questions have potentially important implications for mitigating the effects of hearing loss *via* devices such as cochlear implants.

## 1. Introduction

Pitch—the perceptual correlate of acoustic repetition rate or fundamental frequency (F0)—plays a critical role in both music and speech perception (Plack et al., 2005). Pitch is also thought to be crucial for source segregation—our ability to selectivity hear out and attend to one sound (e.g., a singer or your conversation partner) in the presence of other sounds (e.g., backing instruments or neighboring conversations). Experimental approaches to understanding pitch can be traced back to Seebeck (1841), Ohm (1843), and Helmholtz (1885/1954). Indeed, an early dispute (Turner, 1977) foreshadowed a long-running debate that continues to this day in various forms on what aspects of sound the auditory system extracts in order to derive pitch.

## 2. A time and a place for pitch

### 2.1. Historical roots

The classic pitch-evoking stimulus is a harmonic complex tone, which repeats at the fundamental frequency (F0) and consists of pure tones with frequencies at integer multiples of the F0 (F0, 2F0, 3F0, etc.). The components that form the harmonic tone complex are known as harmonics. We perceive a pitch corresponding to the F0 of a harmonic complex tone, even when the component at F0 itself is missing (the so-called pitch of the missing fundamental; Oxenham, 2012). Much of the debate surrounding pitch has focused on whether pitch is extracted *via* the frequency-to-place mapping that occurs along the basilar membrane (place code; e.g., Wightman, 1973; Terhardt, 1974; Cohen et al., 1995), *via* the timing of stimulus-driving spiking activity in the auditory nerve that is phase-locked to the periodicities present in the stimulus (temporal or time code; Licklider, 1951; Cariani and Delgutte, 1996; Meddis and O’Mard, 1997), or *via* some combination of the two (place-time code; Shamma and Klein, 2000; Cedolin and Delgutte, 2010).

Place theories can be likened to a Fourier transform, followed by pattern recognition or template matching to identify the F0 based on the pattern of places along the basilar membrane responding to different harmonics of a complex tone. These theories or models are often referred to as rate-place models, because they are based on the average firing rate and the tonotopic location of auditory-nerve fibers. Time theories have often been implemented *via* an autocorrelation function, again with either a peak-picking or template-matching stage to identify the dominant underlying periodicity. This timing information can be extracted from the temporal fine structure (TFS) of individual spectrally resolved harmonics, as well as from the temporal envelope fluctuations at the F0 produced by the interactions of spectrally unresolved harmonics (Oxenham, 2012). The contrast between the spectral representation and the autocorrelation function goes some way toward explaining why it has been so difficult to distinguish between the two approaches: the power spectral density and the autocorrelation functions are Fourier transforms of each other, meaning that they are mathematically equivalent and any change to one representation will invariably lead to a change in the other.

Aside from being difficult to distinguish between peripheral rate-place and time codes, the question becomes moot by the level of the cortex, because neurons no longer phase-lock to frequencies higher than a few hundred hertz, meaning that any code based on phase-locked information must have been transformed to another code by this stage of processing (Fishman et al., 2013). So why should we be interested in how information is being extracted from the auditory periphery? One strong rationale is that people with sensorineural hearing loss and/or cochlear implants can be severely limited in their perception of pitch. Understanding how pitch is extracted in the normally functioning auditory periphery may provide important insights into how best to improve pitch perception *via* devices such as cochlear implants.

### 2.2. Rethinking arguments in favor of a time code

A number of arguments exist in favor of a time code for pitch. However, recent work has led to a rethinking of many of these arguments, as listed below.

#### 2.2.1. Pitch is still heard, even in the absence of any place cues

Amplitude-modulated white noise can elicit a pitch (Burns and Viemeister, 1976, 1981), as can a harmonic complex tone that has been highpass filtered to remove any spectrally resolved harmonics (Houtsma and Smurzynski, 1990). The pitch of such sounds is thought to be extracted *via* the periodicity in the temporal envelope of the stimulus, providing *prima facie* evidence that periodic temporal information can be extracted from auditory-nerve activity to encode pitch.

However, *temporal-envelope pitch is fragile.* The resulting pitch is susceptible to interference through noise or reverberation (Qin and Oxenham, 2005), insufficient to convey multiple simultaneous pitches (Carlyon, 1996; Micheyl et al., 2010; Graves and Oxenham, 2019), and produces discrimination thresholds (just-noticeable differences in pitch) that are several times worse than those of complex tones with spectrally resolved harmonics (e.g., Mehta and Oxenham, 2020). This evidence for poor human processing of temporal-envelope pitch suggests that the timing information extracted from the envelope is insufficient to explain the highly salient and accurate perception of pitch we experience with everyday sounds. Indeed, our insensitivity to temporal-envelope pitch poses a problem for timing-based models of pitch, which generally perform too well (relative to human listeners) in cases where only temporal-envelope cues are present (Carlyon, 1998), and require somewhat *ad hoc* assumptions to bring their predictions into line with the perceptual data (Bernstein and Oxenham, 2005; de Cheveigné and Pressnitzer, 2006).

#### 2.2.2. Pitch discrimination is too good to be explained by place cues

We are exquisitely sensitive to small changes in the frequency of pure tones and the F0 of complex tones, to the extent that trained listeners can detect changes of less than 1% (e.g., Micheyl et al., 2006). A place code requires the change in frequency to produce a detectable change in the response level at one or more places along the basilar membrane (leading to a change in average firing rate in one or more auditory-nerve fibers). Standard estimates of human frequency selectivity (Glasberg and Moore, 1990), combined with estimates of the level change needed to be detectable, lead to predicted thresholds for frequency discrimination and frequency-modulation detection that are considerably higher (worse) than observed in humans (Micheyl et al., 2013). Moreover, computational modeling suggests that the amount of information present in the timing of auditory-nerve fibers can exceed the information present when considering just the spatial distribution of average firing rates by two or more orders of magnitude (Siebert, 1970; Heinz et al., 2001; Guest and Oxenham, 2022).

On the other hand, *place cues may be more accurate than we thought.* Early estimates of peripheral frequency selectivity came from physiological studies in small mammals (e.g., Kiang et al., 1967). More recent work combining otoacoustic emissions with behavioral studies using forward masking has suggested that human cochlear tuning is sharper than that in the most commonly studied smaller mammals by a factor of 2–3 (Shera et al., 2002; Sumner et al., 2018). Sharper tuning implies more accurate place coding of small changes in frequency and pitch. In addition, computational modeling has shown that frequency and intensity discrimination in humans can be explained within the same rate-place framework if the reasonable assumption is made that there exists some non-stimulus-related (noise) correlation between cortical neurons with similar frequency response characteristics (Micheyl et al., 2013; Oxenham, 2018). Finally, the ability to detect small fluctuations in the frequency of pure tones (frequency modulation, or FM) shows a significant correlation with estimates of cochlear tuning in people with a wide range of hearing losses, consistent with expectations based on place-based frequency and pitch coding (Whiteford et al., 2020). Based on these newer results, there may no longer be a need to postulate an additional timing-based code to account for human frequency and pitch sensitivity.

#### 2.2.3. Pitch perception degrades at high frequencies

Our ability to discriminate small changes in the frequency of pure tones degrades at frequencies beyond about 4 kHz (Moore, 1973; Moore and Ernst, 2012), as does our ability to recognize even well-known melodies (Attneave and Olson, 1971). This degradation is at least qualitatively consistent with the loss of phase-locking at frequencies beyond 1–2 kHz observed in other mammalian species, such as cat or guinea pig, and possibly humans (Verschooten et al., 2018). In contrast, the sharpness of cochlear filtering, on which place coding depends, actually improves with increasing frequency (Shera et al., 2002), leading to predictions of better, not worse, pitch discrimination.

However, *changes in pitch at high frequencies may not be due to loss of phase locking.* Several recent strands of evidence suggest that the link between poor high-frequency pitch and degraded phase-locking may not be so clear cut. First, complex pitch perception remains accurate even when spectrally resolved harmonics are all above 8 kHz (and so likely beyond the range of usable phase-locking), so long as the F0 itself remains within the musical pitch range (Oxenham et al., 2011; Lau et al., 2017). This suggests that phase-locked information is not necessary for complex pitch perception. Second, the degradation of frequency and FM sensitivity at high frequencies (and at fast FM rates), which had been ascribed to a loss of usable phase-locked information (Moore and Sek, 1996), is also found for tasks that do not involve TFS but instead involve comparisons of level fluctuations across frequency, as would be needed by a rate-place code for frequency (Whiteford et al., 2020). It may be that sensitivity to frequency changes and pitch at high frequencies is poorer due to cortical, rather than peripheral, limitations because pitch from high frequencies is less common and less relevant to us for everyday communication (Oxenham et al., 2011).

#### 2.2.4. The time code is robust to changes in sound level

Perhaps the most compelling remaining argument is that place cues may be dependent on overall sound level, with cochlear tuning broadening and most auditory-nerve responses saturating at high levels, whereas timing cues are generally less susceptible to non-linearities and saturation (Carney et al., 2015).

However, *human data show level dependencies too.* Behavioral studies show a decrease in the number of spectrally resolved harmonics, and a concomitant decrease in pitch discrimination ability, with increasing sound level, in line with the predicted effects of broader cochlear tuning (Bernstein and Oxenham, 2006a). Also, high-threshold, low-spontaneous-rate auditory-nerve fibers remain unsaturated, even at high sound levels (Liberman, 1978; Winter et al., 1990), leaving open the possibility of rate-place coding over a wide range of sound levels.

In summary, none of the primary arguments in support of phase-locked encoding of TFS cues for pitch remains compelling in light of recent empirical data and computational modeling. Indeed, several aspects of the human data, such as the inability to use timing information when it is presented to the “wrong” place along the cochlea (Oxenham et al., 2004) and the ability to perceive complex pitch with only high-frequency components for which little or no timing information can be extracted (Oxenham et al., 2011; Lau et al., 2017; Mehta and Oxenham, 2022), suggest that timing information may be neither necessary nor sufficient for the perception of pitch.

## 3. Asking why as well as how: Machine learning approaches

As noted in the previous section, it has been suggested that poorer pitch discrimination for high-frequency pure tones may be a consequence of less exposure and less ecological relevance of these high-frequency stimuli, rather than a consequence of poorer peripheral encoding (Oxenham et al., 2011). A more comprehensive approach to ecological relevance was taken earlier by Schwartz and Purves (2004), who suggested that many aspects of pitch perception could be explained in terms of the statistics of periodic sounds in our environment, such as voiced speech. This approach can be thought of as asking “why” pitch perception is the way it is, rather than “how” it is represented in the auditory system. A similar approach has been taken more recently by harnessing deep neural networks (DNN) and training them on a large database of over 2 million brief segments of periodic sounds, taken from speech and music recordings embedded in noise (Saddler et al., 2021). Using a well-established computational model of the auditory periphery (cochlea and auditory nerve) as a front end (Bruce et al., 2018), Saddler et al. (2021) found that after training the networks to identify the F0 of these sounds, the networks were able to reproduce a number of “classical” pitch phenomena, supporting the idea of Schwartz and Purves (2004) that many aspects of pitch perception can be explained in terms of the statistics of the sounds we encounter, and extending it by providing quantitative comparisons of the model’s predictions and human performance.

Saddler et al.’s approach also extended beyond the “why” and returned to “how” by testing the relative importance of the spectral resolution and phase-locking in their front-end model. Their simulation results suggested that the spectral resolution of their model was not critical to their results, but that phase-locking was. This result, taken at face value, might suggest support for time over place models of pitch. However, the predictions are at odds with empirical data showing that poorer spectral resolution, either *via* hearing loss in humans (Bernstein and Oxenham, 2006b) or *via* broader cochlear filters in other species (Shofner and Chaney, 2013; Walker et al., 2019), does in fact affect pitch perception. This mismatch between model predictions and empirical data may be because the model has complete access to all the timing information in the simulated auditory nerve. In that sense, the conclusion from the DNN model can be treated as a restatement of the earlier findings from optimal-detector or ideal-observer models (Siebert, 1970; Heinz et al., 2001) that timing information from the auditory nerve provides much greater coding accuracy than average firing rate (rate-place code), and so is more likely to influence model performance. Although the DNN approach holds great promise, the implementations so far have not been tested on the most critical pitch conditions (e.g., on spectrally resolved harmonics outside the range of phase locking) and have remained limited to F0s between 100 and 300 Hz. Although this range spans the average F0s of male (∼100 Hz) and female (∼200 Hz) human voices, it represents less than 2 of the more than 7-octave range of musical pitch, meaning that the majority of our pitch range remains to be explored with this approach.

## 4. Remaining questions and clinical implications

### 4.1. Why is timing extracted from the temporal envelope but not TFS?

If the auditory system can extract pitch from the temporal envelope, why not from TFS? A speculative reason is based on the processing that occurs in the brainstem and midbrain. Temporal-envelope modulation produces amplitude fluctuations that are broadly in phase across the entire stimulated length of the basilar membrane. Many types of neurons in the brainstem and beyond are known to integrate information from across auditory nerve fibers with a range of characteristic frequencies (CFs). By receiving input from auditory-nerve fibers that are synchronized with the period of the temporal envelope and are in phase with each other, the responses from such neurons can be more highly synchronized to the waveform (in terms of vector strength) than those in the auditory nerve itself (Joris et al., 2004). In the case of responses to the TFS of a sinusoidal component (a pure tone or a spectrally resolved harmonic), however, the rapid phase transition of the traveling wave around CF (Shamma and Klein, 2000) means that even auditory-nerve fibers with similar CFs are unlikely to be in phase with each other. The outcome could therefore be desynchronized input to brainstem units, and an inability to transmit the phase-locked responses to TFS beyond the auditory nerve. Note that some brainstem units, such as the globular and spherical bushy cells in the cochlear nucleus, do show highly phase-locked responses to low-frequency CF tones (Joris et al., 1994). However, these are only more synchronized than the auditory-nerve fibers below about 1 kHz, and drop off rapidly thereafter, a pattern that reflects behavioral sensitivity to binaural timing differences but not to monaural or diotic pitch. One possibility, therefore, is that sensitivity to temporal-envelope periodicity is based on brainstem and midbrain sensitivity and tuning to amplitude modulation (Joris et al., 2004). Perceptual sensitivity to amplitude modulation deteriorates above about 150 Hz (Kohlrausch et al., 2000), also with an upper limit of around 1 kHz (Viemeister, 1979). In contrast, information regarding the frequency components themselves may be based solely on place or tonotopic information. Therefore, the difference between the strong pitch based on low-number spectrally resolved components and high-numbered unresolved components may reflect a difference between rate-place coding of the former and temporal (phase-locked) coding of the latter.

### 4.2. Implications for cochlear implants

Cochlear implants are the world’s most successful sensorineural prosthetic device, providing hearing to over one million people worldwide (Zeng, 2022). Despite their success, cochlear implants do not provide “normal” hearing to their users, and one major shortcoming involves the transmission of pitch. Pitch has been defined in multiple ways for cochlear implants. “Place pitch” refers to the sensation reported by cochlear-implant users as the place of stimulation is changed by altering which electrode is activated (Nelson et al., 1995); “rate pitch” or “temporal pitch” is the sensation reported by cochlear-implant users when the electrical pulse rate is changed (Pijl and Schwarz, 1995; Zeng, 2002). For pure tones in acoustic hearing, place and rate covary, but for complex tones, they can be dissociated and are typically referred to as pitch (corresponding to the F0) and brightness (an aspect of timbre related to the spectral centroid of the stimulus). The rate pitch experienced by cochlear-implant users is most akin to the temporal-envelope pitch experienced by normal-hearing listeners in the absence of spectrally resolved harmonics (Carlyon et al., 2010; Kreft et al., 2010), whereas cochlear-implant place pitch seems to behave more like brightness in normal-hearing listeners than pitch (Allen and Oxenham, 2014).

The type of pitch that is not available to cochlear-implant users with current devices is the one that normal-hearing listeners rely on: the salient pitch provided by low-numbered, spectrally resolved harmonics. Some efforts have been made to provide this information to cochlear-implant users *via* TFS cues, but while there may be benefits to binaural hearing (Francart et al., 2015), there is no evidence yet to suggest that pitch salience or accuracy comparable to that in normal-hearing listeners can be induced *via* temporal coding (Landsberger, 2008; Kreft et al., 2010; Magnusson, 2011). The failure to induce accurate pitch perception *via* electrical pulse timing is expected, if we accept that pitch is typically conveyed *via* place cues, and that timing cues can only elicit the relatively crude pitch normally produced by temporal-envelope cues. Would it be possible to provide cochlear-implant users with sufficiently accurate place cues to recreate the kind of pitch elicited *via* spectrally resolved harmonics? Recent studies using acoustic vocoder simulations suggest that this will not be possible with current technology (Mehta and Oxenham, 2017; Mehta et al., 2020). These studies suggest that the spectral resolution required to transmit resolved harmonics requires the equivalent of filter slopes that exceed 100 dB/octave. Current cochlear implants have resolution that seems equivalent to slopes somewhere between 6 and 12 dB/octave (Oxenham and Kreft, 2014), perhaps extending to 24 dB/octave when using focused stimulation techniques (DeVries and Arenberg, 2018; Feng and Oxenham, 2018). Thus, the unfortunate conclusion is that the limited spectral resolution of cochlear implants is unlikely to provide the information necessary to elicit a salient pitch. This conclusion provides an additional impetus for the search for new technologies, based perhaps on neurotrophic agents to decrease the distance between electrodes and neurons, a different stimulation site, such as the auditory nerve, or a different stimulation strategy based, for instance, on optogenetic technology (Oxenham, 2018).

## Data availability statement

The original contributions presented in this study are included in this article/supplementary material, further inquiries can be directed to the corresponding author.

## Author contributions

AO conceived and carried out the work and approved the submitted version.
